# Design, synthesis, and biological evaluation of arylmethylpiperidines as Kv1.5 potassium channel inhibitors

**DOI:** 10.1080/14756366.2021.2018683

**Published:** 2022-01-10

**Authors:** Lingyue Zhao, Qian Yang, Yiqun Tang, Qidong You, Xiaoke Guo

**Affiliations:** a Jiang Su Key Laboratory of Drug Design and Optimization, China Pharmaceutical University, Nanjing, China; b Department of Medicinal Chemistry, School of Pharmacy, China Pharmaceutical University, Nanjing, China; c Department of Clinical Pharmacy, School of Basic Medical Sciences and Clinical Pharmacy, China Pharmaceutical University, Nanjing, China

**Keywords:** Kv1.5 inhibitors, atrial fibrillation, anti-arrhythmia

## Abstract

Kv1.5 potassium channel, encoded by KCNA5, is a promising target for the treatment of atrial fibrillation, one of the common arrhythmia. A new series of arylmethylpiperidines derivatives based on **DDO-02001** were synthesised and evaluated for their ability to inhibit Kv1.5 channel. Among them, compound **DDO-02005** showed good inhibitory activity (IC_50_ = 0.72 μM), preferable anti-arrhythmic effects and favoured safety. These results indicate that **DDO-02005** can be a promising Kv1.5 inhibitor for further studies.

## Graphical abstract

**Figure:**
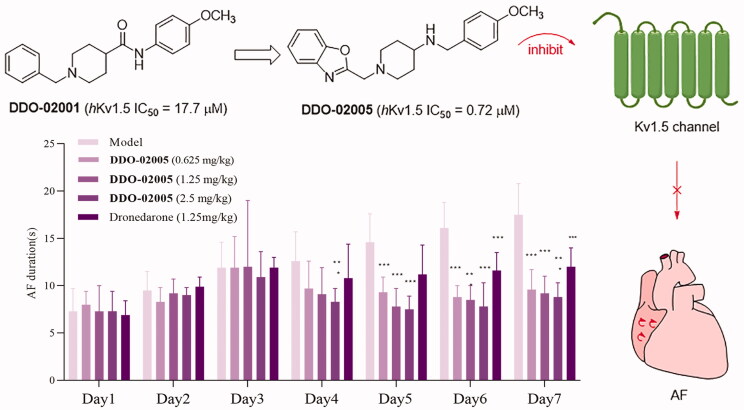

## Introduction

Atrial fibrillation (AF) is one of the most common clinical arrhythmia with a high prevalence in general population^1^, which has a close relationship with other cardiovascular diseases such as stroke, heart failure and ischaemic heart disease^2^^,^^3^. The atria in patients with AF can develop sustained, rapid (400–600 per minute) and irregular impulsion^4^, leading to a reduced quality of life.

An important mechanism for AF is atrial electrical remodelling, which is characterised by significant shortening of atrial effective refractory period (ERP) and action potential duration (APD)^5^^,^^6^ accompanied by prolonged ventricular conduction. This pathological phenomenon is triggered by the weakening of ultra-rapid delayed rectifier potassium current (*I*_Kur_) through ultra-rapid delayed rectifier potassium channel encoded by KCNA5 (Kv1.5) gene^7–9^, which is only expressed in atria^10^^,^^11^. Scientists have discovered that over-expression of Kv1.5 reconstituted a 4-aminopyridine-sensitive outward K^+^ current, shortened the action potential duration, eliminated early after depolarisations, shortened the QT interval, decreased dispersion of repolarisation, and increased the heart rate^6^. The underlying therapeutic principle seems clear that Kv1.5 current suppression is expected to lead to an extension in APD and increase the ERP of fibrotic atria^12^^,^^13^.

**Figure 1.**
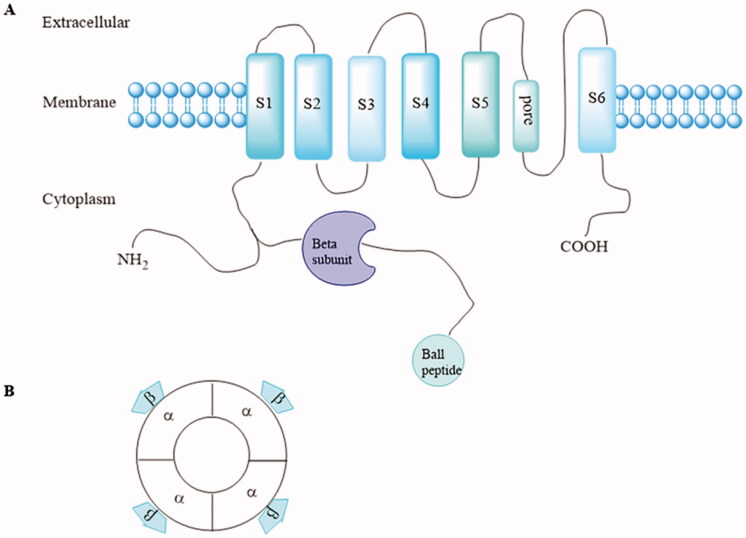
(A) Structure of one Kv1.5 *α*-subunit with six membrane-spanning domains and the intracytoplasmic accessory *β*-subunits. (B) *α* and accessory *β*-subunits co-assemble as tetramers to form the functional channel.

Kv1.5 channel contains eight subunits, including four identical pore-forming *α*-subunits encoded by the KCNA5 gene and four accessory *β*-subunits (Kv*β*1.2, Kv*β*1.3, and Kv*β*2.1) that bind to the *N*-terminus of the *α*-subunit to form *α*4*β*4 complexes. Every *α*-subunit contains six transmembrane-spanning segments (S1–S6) with cytoplasmic *N*-and *C*-terminal domains (Figure 1)^14^. The *β*-subunit field received a major boost when rKv*β*1.1 and rKv*β*2.1 from ratbrain cDNA libraries were isolated by bovine amino acid sequence^14^^,^^15^. Since then, multiple K^+^ channel *β-*subunit genes such as Kv*β*3.1, Kv*β*4.1, Kv*β*1.2, Kv*β*1.3 were cloned from brain and cardiac resources, respectively^16–18^. Kv*β*1.1–1.3 proteins arise by alternative splicing from the same gene^17^, whereas Kv*β* 2.1, 3.1, and 4.1 are derived from distinct genes. Though multiple K^+^ channel *β* subunit genes were encoded by different genes, they shared a common conserved core of over 85% amino identity, which laid a foundation for interaction with Kv*α* subunits. Phosphorylation of the *β-*subunit is important in modulation of *α*/*β* interactions^11^. In Kv1.5 channel, Kv1.5 *α*-subunits co-assembled with Kv*β*1.2 subunits to form the *I*_Kur_ in human atrium^19^.

**Figure 2.**
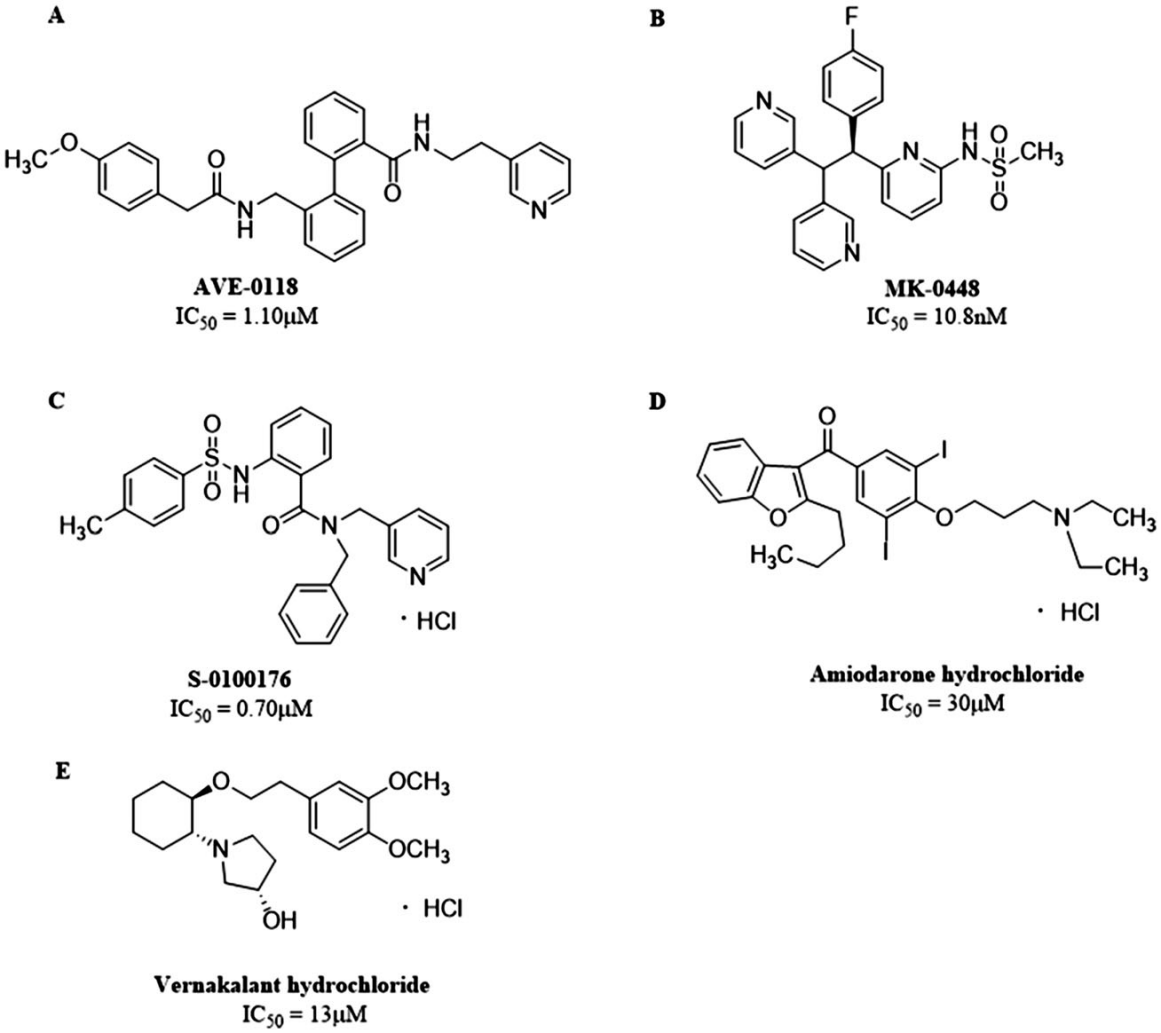
Structures of (A) **AVE-0118**. (B) **MK-0448**. (C) **S-0100176**. (D) **Amiodarone hydrochloride**. (E) **Vernakalant hydrochloride**.

So far, two categories of Kv1.5 inhibitors have been discovered: selective inhibitors, including **AVE-0118**, **MK-0448**, **S-0100176**, etc., and non-selective inhibitors such as **Amiodarone**, **Vernakalant** and so on (Figure 2). The selectivity of *I*_Kur_ blockers to prolong atrial versus ventricular ERP can be explained by the presence of *I*_Kur_ in atria. However, in healthy non-remodeled atria, the prolongation of AERP can be related to the blockade of *I*_Na_. Most of the new drugs developed as selective *I*_Kur_ blockers show mixed ion channel activity, blocking other cardiac K^+^, Ca^2+^ and Na^+^ currents, and their affinity is comparable to *I*_Kur_, that is, they produce non-selectivity *I*_Kur_ blockade^19^. For instance, Vernakalant inhibits the *I*_Na_ and slow conduction velocity and increase diastolic threshold of excitation in atria but not in ventricles^20–22^, and some *I*_Kur_ blockers such as **AVE-0118** inhibits *I*_to_ to prolong human APD at the plateau level^23^. Therefore, drugs that affect multiple currents can be much more effective than blockers that affect only one current, as several different ionic currents contribute to human atrial AP^24^.

**Figure 3.**
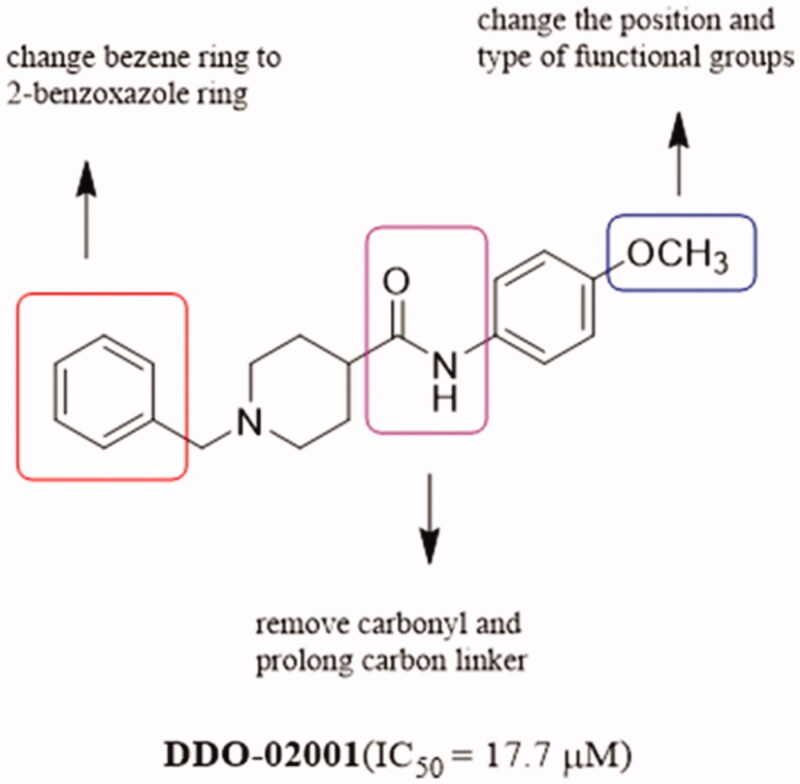
Structure of DDO-02001 and strategy for optimisation.

In order to discover Kv1.5 inhibitors with new scaffold, an in-house compound library was screened using whole-patch clamp technique. Compound **DDO-02001** with moderate inhibitory effect on Kv1.5 channel (IC_50_ = 17.7 μM) was obtained as a lead compound. To improve the Kv1.5 inhibitory activity, a series of compounds with arylmethylpiperidines skeleton were designed (Figure 3) and synthesised, their biological activities were tested and structure-activity relationships (SAR) were discussed. Ultimately, the potent Kv1.5 channel inhibitor **DDO-02005** was acquired with acceptable therapeutic effect in atrial fibrillation model and considerable inhibitory effect on arrhythmia, herein the arrhythmia was induced by aconitine in rats.

## Experimental

### Chemistry

All starting materials and solvents were purchased from commercial sources and used without any additional purification. Melting points (m.p.) were detected by a Melt-Temp II apparatus. All the reactions were monitored using TLC silica gel plates (GF254, 0.25 mm) and visualised under UV light. The ^1^H-NMR and ^13^C-NMR spectra were measured on a Bruker AV-300 instrument using deuterated solvents with tetramethylsilane (TMS) as internal standard. High-resolution mass spectra (HRMS) were obtained on Water Q-TOF micro mass spectrometer. The purity (≥95%) of the target compounds was verified by High Performance Liquid Chromatography (HPLC) analysis (Agilent C18 column, 4.6 × 150 mm, 5 μm).

#### Synthesis of tert-butyl4-((4-methoxyphenyl)carbamoyl)piperidine-1-carboxylate (2)

To a solution of 1-(*tert*-butoxy carbonyl) piperidine-4-carboxylic acid (1.29 g, 0.01 mol) in anhydrous THF (10 ml), EDC (0.01 mol) and DMAP (0.01 mol) were added. The mixture was stirred at r.t. for 15 min. Then 4-methoxyaniline (0.011 mol) was added. The mixture was poured into water and filtered after stirring for another 1 h at r.t., then the solid was washed with water and dried under infra-red light to give white solid. (1.5 g, 45%, m.p. 154–155 °C); ^1^H-NMR (300 MHz, CDCl_3_) *δ* 7.40 (d, *J* = 9 Hz, 2H), 7.26 (s, 1H), 6.85 (d, *J* = 9 Hz, 2H), 4.16 − 4.21 (m, 2H), 3.79 (s, 3H), 2.74 − 2.83 (m, 2H), 2.32 − 2.36 (m, 1H), 1.88 − 1.92 (m, 2H), 1.59 − 1.80 (m, 2H), 1.47 (s, 9H).

#### Synthesis of N-(4-methoxyphenyl) piperidine-4-carboxamide trifluoroacetate (3)

To a solution of *tert*-butyl 4–(3-methoxyphenylcarbamoyl) piperidine-1-carboxylate (3.34 g, 0.01 mol) in methanol (40 ml), trifluoroacetic acid (TFA) (1.5 ml, 0.02 mol) was added. The mixture was stirred at room temperature for 12 h and concentrated in vacuum until dryness. The solid was washed with diethyl ether and dried under infra-red light to give white solid (1.5 g, 43.1%); ^1^H-NMR (300 MHz, CDCl_3_) *δ* 9.71 (s, 1H), 7.53 − 7.47 (m, 2H), 6.91 − 6.86 (m, 2H), 3.78 (s, 3H), 3.09 (dd, *J* = 12.4, 7.1, 5.3 Hz, 2H), 2.99 (dd, *J* = 12.4, 5.4 Hz, 2H), 3.13 − 3.03 (m, 2H), 3.05 − 2.95 (m, 2H), 1.96 (dd, *J* = 12.4, 7.1 Hz, 2H), 1.85 (dd, *J* = 12.3, 7.0 Hz, 2H).

#### Synthesis of 1-(benzo[d]oxazol-2-ylmethyl) piperidin-4-one (5)

To a solution of peridin-4-one hydrochloride (3.96 g, 0.04 mol) and K_2_CO_3_ (5 g, 0.036 mol) in CH_3_CN (80 ml), 2-(chloromethyl) benzo[*d*]oxazole (5.85 g, 0.035 mol) in CH_3_CN (50 ml) was added. The mixture was refluxed for 4 h and filtered. The solvent was evaporated until dryness. The residue was purified by column chromatograph over silica gel (EA/PE 1:2). The pure fractions were collected, and the solvent was evaporated (4.8 g, 60%, m.p. 86–88 °C); ^1^H-NMR (300 MHz, CDCl_3_) *δ* 7.70 − 7.73 (m, 1H), 7.52 − 7.57 (m, 1H), 7.32 − 7.39 (m, 2H), 4.02 (s, 2H), 2.98 (t, *J* = 6 Hz, 4H), 2.54 (t, *J* = 6 Hz, 4H).

#### Synthesis of 1-benzyl-N-(4-methoxyphenyl) piperidine-4-carboxamide (DDO-02001)

To a solution of **3** (1 g, 0.0028 mol) and K_2_CO_3_ (5 g, 0.036 mol) in acetonitrile, (bromomethyl)benzene (0.7 ml, 0.0055 mol) was added. After refluxing for 2 h, the mixture was poured into water and extracted by ethyl acetate (20 ml*3). The organic layer was then separated and dried with anhydrous sodium sulphate, filtered and concentrated. Then purified by column chromatography over silica gel (EA/PE l:1) to provide crude product. The crude product was crystallised in ethyl acetate to give white solid (0.6 g, 66%, m.p. 157–159 °C); ^1^H-NMR (300 MHz, CDCl_3_) *δ* 7.49 − 7.41 (m, 2H), 7.37 (d, *J* = 4.4 Hz, 4H), 7.16 (s, 1H), 6.89 (d, *J* = 8.9 Hz, 2H), 3.83 (s, 3H), 3.57 (s, 2H), 3.03 (d, *J* = 10.9 Hz, 2H), 2.26 (d, *J* = 10.9 Hz, 1H), 2.08 (d, *J* = 11.1 Hz, 2H), 2.02 − 1.83 (m, 4H). ^13^C-NMR (75 MHz, DMSO-d_6_) *δ* 168.86, 151.62, 133.61, 126.42, 124.40, 123.54, 122.35, 117.24, 109.36, 72.84, 72.41, 71.99, 58.50, 50.77, 48.33, 39.46, 24.28. HRMS (ESI): calcd for *m/z* C_20_H_24_N_2_O_2_ [M + H]^+^ 325.19105, found 325.19184. HPLC (methanol: water = 80: 20): *t*_R_=3.1 min, 99.43%.

#### Synthesis of 1-(benzo[d]oxazol-2-ylmethyl)-N-(4-methoxyphenyl) piperidine-4-carboxamide (DDO-02002)

To a solution of **3** (1 g, 0.0028 mol) and K_2_CO_3_ (5 g, 0.036 mol) in acetonitrile, 2-(chloromethyl)benzo[*d*]oxazole (0.918 g, 0.0055 mol) was added, the mixture was refluxed for 2 h, poured into water, extracted by ethyl acetate, dried over Na_2_SO_4_, and filtered, and the solvent was evaporated until dryness. The residue was purified by column chromatography over silica gel (EA/PE l:1). The solvent was evaporated and the fraction was crystallised in ethyl acetate. The precipitate was filtered off and dried to give white solid (0.7 g, 68%, m.p. 190–194 °C); ^1^H-NMR (300 MHz, DMSO-d_6_) *δ* 9.72 (s, 1H), 7.77 (dt, *J* = 7.8, 1.6 Hz, 2H), 7.59 − 7.46 (m, 2H), 7.45 − 7.35 (m, 2H), 6.88 (d, *J* = 9.0 Hz, 2H), 3.90 (s, 2H), 3.73 (s, 3H), 3.00 (d, *J* = 10.9 Hz, 2H), 2.41 − 2.09 (m, 3H), 1.86 − 1.61 (m, 4H). HRMS (ESI): calcd. for *m/z* C_20_H_24_N_2_O_2,_ [M + H]^+^ 366.18122, found 366.18188. HPLC (methanol: water = 80: 20): *t*_R_=5.9 min, 95.09%.

#### General procedure for the synthesis of DDO-02003-DDO-02009

To a solution of **5** (0.1 g, 0.434 mmol), 6–17 (0.651 mmol) in CH_2_Cl_2_ were added. NaB(OAc)_3_H (0.183 g, 0.868 mol) was added to the solution and stirred at room temperature. After the reaction, the solvent was evaporated until dryness and quenched by adding saturated NH_4_Cl. Then product was extracted with CH_2_Cl_2_ (20 ml*3) and dry in vacuum. HCl-EA solution was added to form the hydrochloride salt, then the solid was recrystallised with EA.

The yield, melting point, analytical data, and spectral data of each compound are given below.

##### 1-(benzo[d]oxazol-2-ylmethyl)-N-(4-methoxyphenyl) piperidin-4-amine dihydrochloride (DDO-02003)

0.067 g, 46%, white powder, m.p. >250*°*C; ^1^H-NMR (300 MHz, D_2_O) *δ* 7.72 (dd, *J* = 7.4, 1.8 Hz, 1H), 7.50 − 7.37 (m, 1H), 7.50 − 7.37 (m, 2H), 7.29 (dd, *J* = 7.9, 5.4 Hz, 2H), 7.07 − 7.01 (m, 2H), 4.70 (s, 2H), 3.80 (d, *J* = 3.9 Hz, 1H), 3.76 (s, 3H), 3.75 (s, 2H), 3.26 (td, *J* = 13.2, 2.9 Hz, 2H), 2.27 (d, *J* = 13.9 Hz, 2H), 2.11 − 1.92 (m, 2H). HRMS (ESI) calcd. for *m/z* C_20_H_23_N_3_O_2_, [M + H]^+^ 338.18639, found 338.1863. HPLC (methanol*:* water = 80*:* 20): *t*_R_=6.9 min, 95.18%.

##### 1-(benzo[d]oxazol-2-ylmethyl)-N-benzylpiperidin-4-amine dihydrochloride (DDO-02004)

0.058 g, 42%; white powder, m.p. > 250 °C; ^1^H-NMR (300 MHz, Deuterium Oxide) *δ* 7.75 − 7.68 (m, 1H), 7.65 − 7.59 (m, 1H), 7.43 (dd, *J* = 7.5, 1.5 Hz, 2H), 7.38 (s, 5H), 4.70 (s, 2H), 4.21 (s, 2H), 3.80 (d, *J* = 12.8 Hz, 2H), 3.67 − 3.42 (m, 1H), 3.28 (t, *J* = 12.9 Hz, 2H), 2.44 (d, *J* = 14.0 Hz, 2H), 2.17 − 1.80 (m, 2H). HRMS (ESI) calcd. for *m/z* C_20_H_23_N_3_O, [M + H]^+^ 322.19166, found 322.19139. HPLC (methanol*:* water = 80*:* 20): *t*_R_ = 4.5 min, 98.09%.

##### 1-(benzo[d]oxazol-2-ylmethyl)-N-(4-methoxybenzyl)piperidin-4-amine dihydrochloride (DDO-02005)

0.06 g, 40%; white powder, m.p. 207–211*°*C; ^1^H-NMR (300 MHz, Deuterium Oxide) *δ* 7.55 (dd, *J* = 18.2, 7.4 Hz, 2H), 7.41 − 7.18 (m, 4H), 6.90 (d, *J* = 8.2 Hz, 2H), 4.07 (s, 2H), 3.81 (s, 2H), 3.70 (s, 3H), 3.21 − 2.86 (m, 2H), 2.24 (t, *J* = 12.0 Hz, 2H), 2.14 − 1.97 (m, 2H), 1.72 − 1.43 (m, 2H). HRMS (ESI) calcd. for *m/z* C_21_H_25_N_3_O_2_, [M + H]^+^ 352.20108, found 352.20195. HPLC (methanol*:* water = 80*:* 20): *t*_R_ = 4.3 min, 95.27%.

##### 1-(benzo[d]oxazol-2-ylmethyl)-N-(4-fluorobenzyl) piperidin-4-amine dihydrochloride (DDO-02006)

0.065 g, 44%; white powder, m.p. >250 °C; ^1^H-NMR (300 MHz, Methanol-d_4_) *δ* 7.83 − 7.77 (m, 1H), 7.75 − 7.68 (m, 1H), 7.64 (dd, *J* = 8.5, 5.3 Hz, 2H), 7.50 (dd, *J* = 8.3, 7.1, 3.9 Hz, 2H), 7.25 (t, *J* = 8.6 Hz, 2H), 4.54 (s, 2H), 4.32 (s, 2H), 3.70 (d, *J* = 12.1 Hz, 2H), 3.50 (d, *J* = 12.3 Hz, 1H), 3.08 (t, *J* = 12.0 Hz, 2H), 2.44 (d, *J* = 13.2 Hz, 2H), 2.19 − 2.04 (m, 2H). HRMS (ESI) calcd. for m/z C_20_H_22_FN_3_O, [M + H]^+^ 340.18197, found 340.18205. HPLC (methanol: water = 80: 20): t_R_ = 11 min, 96.98%.

##### 1-(benzo[d]oxazol-2-ylmethyl)-N-(2-methoxybenzyl)piperidin-4-amine dihydrochloride (DDO-02007)

0.068 g, 45%; white powder, m.p. >250 °C; ^1^H-NMR (300 MHz, Deuterium Oxide) *δ* 7.77 − 7.70 (m, 1H), 7.68 − 7.62 (m, 1H), 7.52 − 7.35 (m, 3H), 7.31 (dd, *J* = 7.5, 1.7 Hz, 1H), 7.09 − 6.92 (m, 2H), 4.71 (s, 2H), 4.24 (s, 2H), 3.82 (s, 5H), 3.62 − 3.47 (m, 1H), 3.30 (dd, *J* = 13.8, 11.0 Hz, 2H), 2.45 (d, *J* = 14.0 Hz, 2H), 2.11 − 1.90 (m, 2H). HRMS (ESI) calcd, for *m/z* C_21_H_25_N_3_O_2_, [M + H]^+^ 352.20329, found 352.20195. HPLC (methanol: water = 80: 20): *t*_R_ = 6.13 min, 96.50%.

##### 1-(benzo[d]oxazol-2-ylmethyl)-N-(4-methylbenzyl)piperidin-4-amine dihydrochloride (DDO-02008)

0.070 g, 48%; white powder, m.p. 205–207*°*C; ^1^H-NMR (300 MHz, Methanol-d_4_) *δ* 7.77 − 7.71 (m, 1H), 7.69 − 7.61 (m, 1H), 7.50 − 7.42 (m, 2H), 7.39 (d, *J* = 7.9 Hz, 2H), 7.29 (d, *J* = 7.7 Hz, 2H), 4.14 (s, 2H), 3.97 (s, 2H), 3.16 (d, *J* = 12.3 Hz, 2H), 3.10 − 3.01 (m, 1H), 2.36 (d, *J* = 11.2 Hz, 2H), 2.17 (d, *J* = 12.3 Hz, 2H), 1.95 (s, 3H), 1.86 − 1.69 (m, 2H). HRMS (ESI) calcd. for *m/z* C_21_H_25_N_3_O, [M + H]^+^ 336.20704, found 336.20686. HPLC (methanol*:* water = 80*:* 20): *t*_R_=5.74 min, 95.79%.

##### 1-(benzo[d]oxazol-2-ylmethyl)-N-phenethylpiperidin-4-amine hydrochloride (DDO-02009)

0.067 g, 46%; white powder, m.p. >250 °C; ^1^H-NMR (300 MHz, Deuterium Oxide) *δ* 7.74 (d, *J* = 7.6 Hz, 1H), 7.65 (d, *J* = 7.9 Hz, 1H), 7.52 − 7.38 (m, 2H), 7.30 (dt, *J* = 20.8, 7.6 Hz, 5H), 4.70 (s, 2H), 3.79 (d, *J* = 13.0 Hz, 2H), 3.51 (td, *J* = 11.9, 6.0 Hz, 1H), 3.38 − 3.17 (m, 4H), 2.96 (t, *J* = 7.6 Hz, 2H), 2.38 (d, *J* = 14.0 Hz, 2H), 2.01 − 1.89 (m, 2H). HRMS (ESI) calcd. for *m/z* C_21_H_25_N_3_O, [M + H]^+^ 336.20704, found 336.20692. HPLC (methanol: water = 80: 20): *t*_R_=6.58 min, 95.24%.

## Biological evaluation

### Whole-patch clamp assay

The HEK 293 cell line that stably expressed *h*Kv1.5 potassium channel was a kind gift from Dr. Gui-Rong Li (Department of Medicine and Department of Physiology, Li Ka Shing Faculty of Medicine, The University of Hong Kong, Pokfulam, Hong Kong, SAR, China). Transfected HEK 293 cells were maintained at 37 °C in Minimal Eagle Medium (MEM) or Dulbecco's Modified Eagle Medium (DMEM) supplemented with 10% foetal bovine serum, 1% penicillin-streptomycin, 2 mmol/L L-glutamine, 0.1 mmol/L nonessential amino acids, 1 mmol/L sodium pyruvate, and 0.2 mg/mL geneticin (Invitrogen Corporation, Carlsbad, CA, USA). Cells were passaged weekly and used at ≤80% confluence. For electrophysiological recordings, the cells were harvested from the culture dish by trypsinisation, and then washed twice with standard MEM or DMEM and maintained in culture medium at room temperature for later use on the same day.

The whole-cell membrane currents were recorded by the patch-clamp technique, using an EPC-10 double patch-clamp amplifier (HEKA, Pfalz, Germany). Recording pipettes, made from borosilicate glass (1.2 mm, o.d.), pulled with a pipette puller (PIP5, HEKA, Germany), had resistances of between 4 and 6 Ω when filled with the pipette solution. After a giga-seal (>10 GΩ) was obtained, the cell membrane was ruptured by gentle suction to establish the whole-cell configuration. The series resistance was electrically compensated to minimise the capacitive surge on the current recording. Peak current amplitude was determined after baseline correction. Pulse software (HEKA, Pfalz, Germany) was used to generate voltage pulse protocols and to record and analyse data. Compounds were applied at least 5 min after current stabilisation. The data are presented as the mean and standard deviation (mean ± SD). The differences between control levels and the changes caused by the compound application were tested using Student's *t*-test. A value of *p* < 0.05 was considered statistically significant. All experiments were performed at 25 °C.

### CaCl_2_-Ach induced AF model

SD rats (250 ± 20 g) were raised in an environment with a temperature of 20–24 °C and a humidity of 50%, and lighting for 12 h, drink and eat freely, and then anaesthetised with 10% chloral hydrate (Sinopharm Chemical Reagent Co., Ltd, Shanghai, China) (3 ml/kg, i.p.), followed by i.v. administration of CaCl_2_ (10 mg/mL) and acetylcholine (ACh; 66 mg/mL) through the caudal vein (1 ml/kg) once a day for 7 days. A typical AF electrocardiogram (ECG) appeared immediately and recovered to sinus rhythm in the following 10 s. The ECG was recorded for the complete experiment procedure from rat anaesthetisation to the ECG being restored to normal, approximately 50 min for each rat each day (i.e. from Day 1 to Day 7).

On Day 4, rats were randomly divided into five groups (*n* = 10/group): (1) AF model group: repeated prior procedure until day 7, CaCl_2_ (10 mg/mL, i.v.) and ACh (66 mg/mL, i.v.); (2) (3) and (4) **DDO-02005** treatment groups: **DDO-02005** (0.625 mg/kg and 1.25 mg/kg, 2.5 mg/kg, i.p.) combined with CaCl_2_ (10 mg/mL) and ACh (66 mg/mL) (i.v.) from Day 4 to Day 7; (5) dronedarone treatment group: dronedarone (1.25 mg/kg, i.p.) combined with CaCl_2_ (10 mg/mL) and ACh (66 mg/mL) (i.v.) from Day 4 to Day 7. Controls were anaesthetised with 10% chloral hydrate (3 ml/kg, i.p.), followed by physiological saline i.v. for 7 days.

The disappearance of the P wave and appearance of the f wave was determined as the beginning of AF while the end was designated by disappearance of the f wave and the appearance of the regular P wave, e.g., sinus rhythm recovery. Other ECG parameters including heart rate (HR), PR, QRS, and rate-corrected QT_c_ interval (QT_cd_ = QT/(R-R)^1/2^) were also recorded as this study was focussed on HR and rate-corrected QT_c_ interval analysis.

### Aconitine induced arrhythmia model

SD rats (250 ± 20 g) were anaesthetised and the jugular vein was surgically separated. After intubation, rats in each group were given intravenous normal saline (model group), each dose of the compound (0.1 mg/kg, 1 mg/kg, 3 mg/kg, 9 mg/kg). 5 min after administration, the jugular vein was given aconitine at a constant rate (0.20–0.22 ml/min, 1 μg/ml).

The experiment uses the BL-420F biological function test system (Chengdu Taimeng Technology Co., Ltd.) to continuously record the changes of the electrocardiogram of the rats before and after the administration (lead II), and calculate the amount of aconitine injected (μg/100g) when the rats have premature ventricular beats (VP).

### Pharmacokinetics studies

12 beagle dogs (male and female) were taken, randomly divided into two groups equally. The experimental animals were fasted overnight (more than 10 h) 12 h before the experiment. The drug was administered at 7:00 am the next day at a dose of 1 mg/kg by intravenous injection and 1.25 mg/kg by oral administration. The drug was dissolved in 100 ml normal saline and administered by gavage. 3 ml of blood was collected from the vein before gavage (0 min) and 10, 20, 30, 45 min, 1, 1.5, 2, 3, 4, 6, 8, 10, 12 h. After gavage, the plasma was collected and stored at −20 °C for the detection of blood drug concentration, and the accurate time of blood collection was recorded in detail. The test dogs were fasted overnight (more than 10 h) before taking the medicine, and the corresponding preparations were given on an empty stomach at 8:30 the next morning. Blood was collected according to the design time point, put into a test tube, stood for half an hour, centrifuged at 3500 rpm for 15 min, supernatant was taken into a centrifuge tube, frozen at −20 °C for 1 week, and the washout period of cross-medication was 1 week.

### Safety studies

The healthy, male guinea pigs weighing 250–300 g were anaesthetised with urethane and fixed on the operating table. Using needle electrodes to pierce the extremities and subcutaneously on the chest, and the whole heart lead electrocardiogram was recorded. The right jugular vein is separated and cannulated. After 5 min, **DDO-02005**, Azimilide, (3 × 10^−7^, 10^−6^, 3 × 10^−6^, 10^−5^, 3 × 10^−5 ^mol/kg) dissolved in saline were given cumulative injections, each concentration is given in equal volume (1.5 ml/kg). After administration, the BL-420F biological function experiment system recorded synchronous body surface electrocardiogram (adjust control parameter gain G: 1mv, time constant T: 0.1 s, low channel filter 100 Hz, scanning speed 250 ms/div, and start 50 Hz suppression).

ECG analysis: To observe the effects of compounds on the heart rate and QT interval of animal standard II lead ECG. Measure the cardiac cycle of each lead. Each lead continuously measured 6 complete cardiac cycles, and the average value was taken as the QT interval of that lead. The QT interval is measured from the beginning of the QRS wave to the end of the T wave. When the T wave is flat or there is a U wave, the method for judging the end of the T wave is: (1) the intersection of the T wave and the equipotential line; (2) the notch between the T wave and the U wave; (3) the intersection point of the T wave descending notch and the equipotential line; if the T wave is low and it is difficult to determine the end point, the lead is discarded. QT_d_ is the difference between the maximum and minimum QT interval in the measured 12 leads, and the calculation formula is QT_d_=QT_max_-QT_min_. Considering that the QT interval is affected by the heart rate, the Bazett formula is used for heart rate correction to calculate the QT_cd_ after the heart rate correction. The calculation formula is QT_cd_ = QT/(R–R)^1/2^, and the R–R in the formula refers to the R–R interval period. All measurement work is manually measured by the experimenter.

## Result and discussion

### Chemistry

**Scheme 1.**
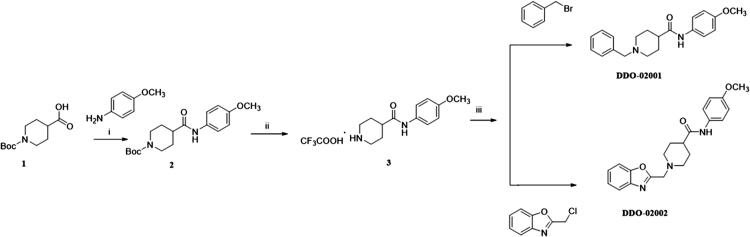
Synthesis of **DDO-02001** and **DDO-02002**. Reagents and conditions: (i) EDCI, DMAP, THF, r.t.; (ii) CF_3_COOH, DMF, r.t.; (iii) K_2_CO_3_, CH_3_CN, 80 °C, reflux.

**Scheme 2.**
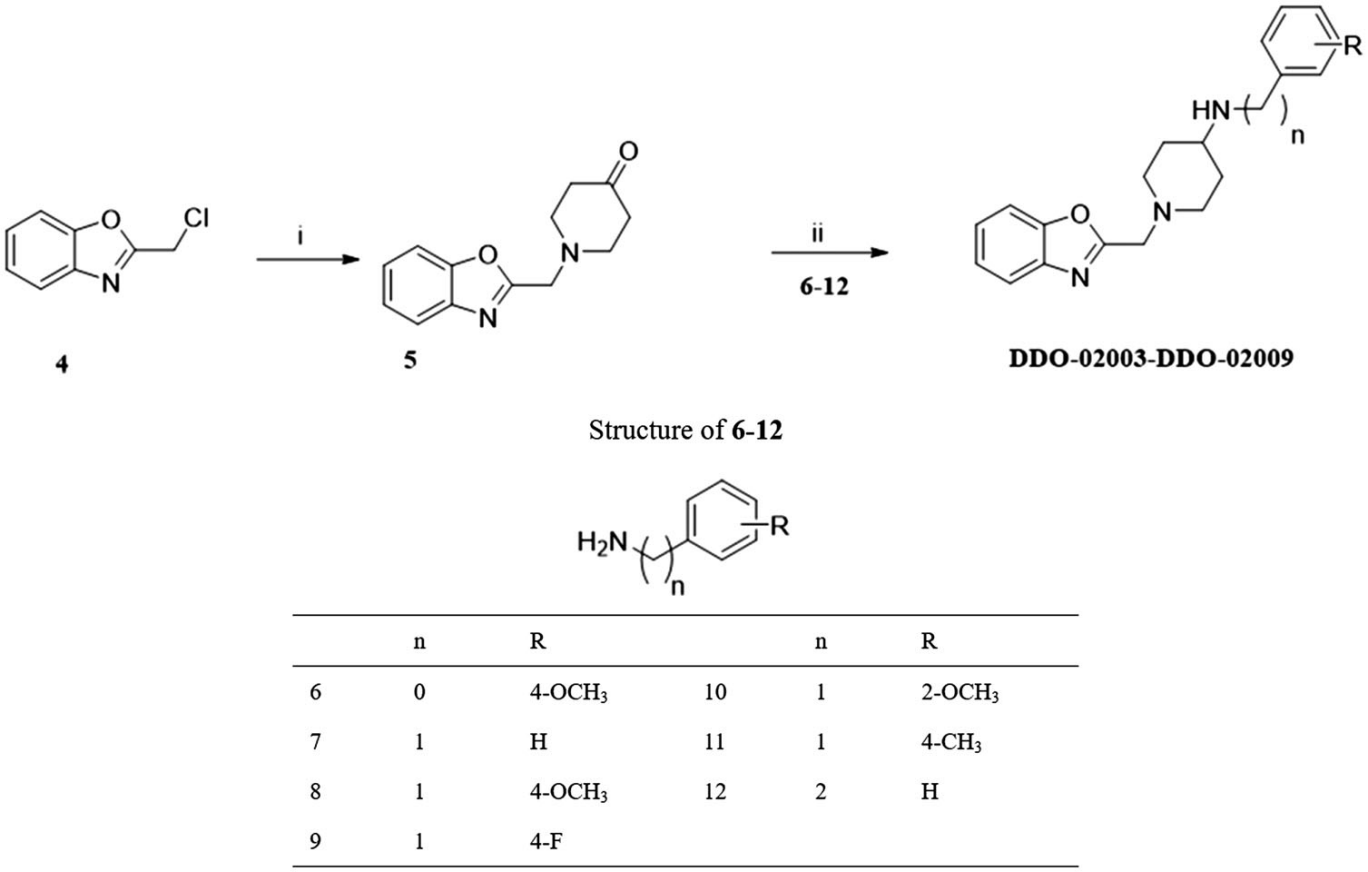
Synthesis of **DDO-02003**-**DDO-02009**. Reagents and conditions: (i) piperidin-4-one-hydrochloride, K_2_CO_3,_ r.t.; (ii) NaB(OAc)_3_H, anhydrous. ClCH_2_CH_2_Cl, r.t.

To improve the Kv1.5 inhibitory activity, we changed the aromatic ring, linker and substituent of **DDO-02001** as shown in Figure 2. As shown in Scheme 1, 1-(tert-butoxycarbonyl)piperidine-4-carboxylic acid (**1**) was reacted with 4-methoxy-aniline, EDCI and DMAP to get **2,** which was then treated with trifluoroacetic acid (TFA) and dichloromethane mixture at room temperature to get secondary amine **3**. **3** was treated with benzyl bromide or 2-(chloromethyl)benzo[d]oxazole in the presence of K_2_CO_3_ to afford **DDO-02001** and **DDO-02002**. Intermediate **5** was synthesised from the reaction of 4 and piperidin-4-one hydrochloride. Treatment of **5** and a series of amine compounds with NaB(OAc)_3_H gave **DDO-02003**～**DDO-02009** as described in Scheme 2.

### Structure-activity relationship studies of arylmethylpiperidines derivatives

In order to investigate the SAR (Structure-activity Relationship), whole-cell patch clamp technique was applied to analyse the inhibitory activities of Kv1.5 channel of target compounds. Results are listed in Table 1.

**Table 1.** Inhibition activities of **DDO-02002-DDO-02009** on Kv1.5 channel 
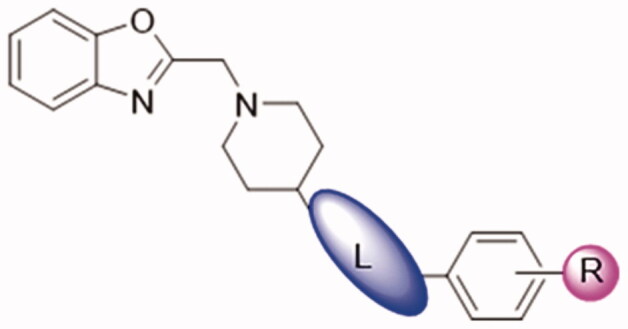

Comp.	L	R	*h*Kv1.5 IC_50_ (μM)
**DDO-02002**	–CONH–	4-OCH_3_	8.96 ± 1.15
**DDO-02003**	–NH–	4-OCH_3_	1.57 ± 0.26
**DDO-02004**	–NH–CH_2_–	H	0.95 ± 0.13
**DDO-02005**	–NH–CH_2_–	4-OCH_3_	0.72 ± 0.08
**DDO-02006**	–NH–CH_2_–	4-F	3.24 ± 0.29
**DDO-02007**	–NH–CH_2_–	2-OCH_3_	>20
**DDO-02008**	–NH–CH_2_–	4-CH_3_	>20
**DDO-02009**	–NH–CH_2_–CH_2_–	H	>20
**DDO-02001**	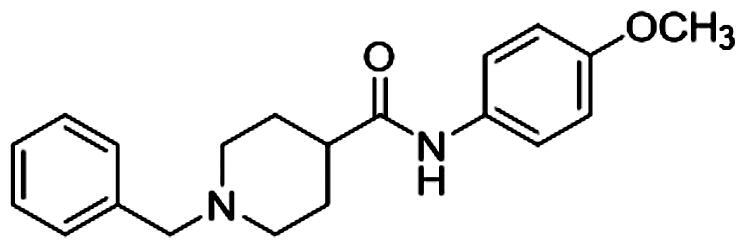		17.71 ± 2.93

By comparing the biological results of compounds **DDO-02001** and **DDO-02002**, it can be seen that replacing the benzene ring of molecule **DDO-02001** with a benzoxazole ring improved the biological activity (**DDO-02001** IC_50_ = 17.7 μM vs. **DDO-02002** IC_50_ = 8.96 μM). Carbonyl is not so important, because the removal of carbonyl lead to the promotion of inhibition effect proved by **DDO-02002** and **DDO-02003** (IC_50_ = 1.57 μM). Replacing the linker L from –NH– to –NH–CH_2_– slightly increased the activity (**DDO-02003** vs. **DDO-02005**); Extending the carbon chain to –NH–CH_2_–CH_2_–, the activity decreased (**DDO-02004** vs **DDO-02009**), suggesting the linker –NH–CH_2_– was the best choice. Different substituents on the benzene ring were changed to discuss their influence on the effect of the compound. Compared with **DDO-02005**, **DDO-02006** with electron withdrawing group on the benzene ring has a decreased inhibition rate of Kv1.5 channel (IC_50_ = 3.24 μM), implying that methoxyl group might be a preferred option to improve the inhibitory activity. Changing the position and type of the electron donating group in **DDO-02005** to get **DDO-02007** and **DDO-02008**, the activity decreased, suggesting that para-methoxy substitution is the best choice to exert inhibitory activity.

Through the above comparison, we screened out the best compound **DDO-02005**, and a series of subsequent experiments were conducted to verify its pharmacodynamic and pharmacokinetic properties.

### Effect of DDO-02005 on AF model induced by CaCl_2_-ACh

A classic pathological model of atrial fibrillation induced by calcium chloride-acetylcholine (CaCl_2_-ACh) in rats was applied to evaluate the therapeutic effect of the active compound **DDO-02005** on atrial arrhythmia as described previously^25^, dronedarone was selected as a positive control. The results were characterised by the changes of atrial fibrillation duration, atrial ERP (AERP) and ventricle ERP (VERP) before and after treatment with **DDO-02005**.

**Figure 4.**
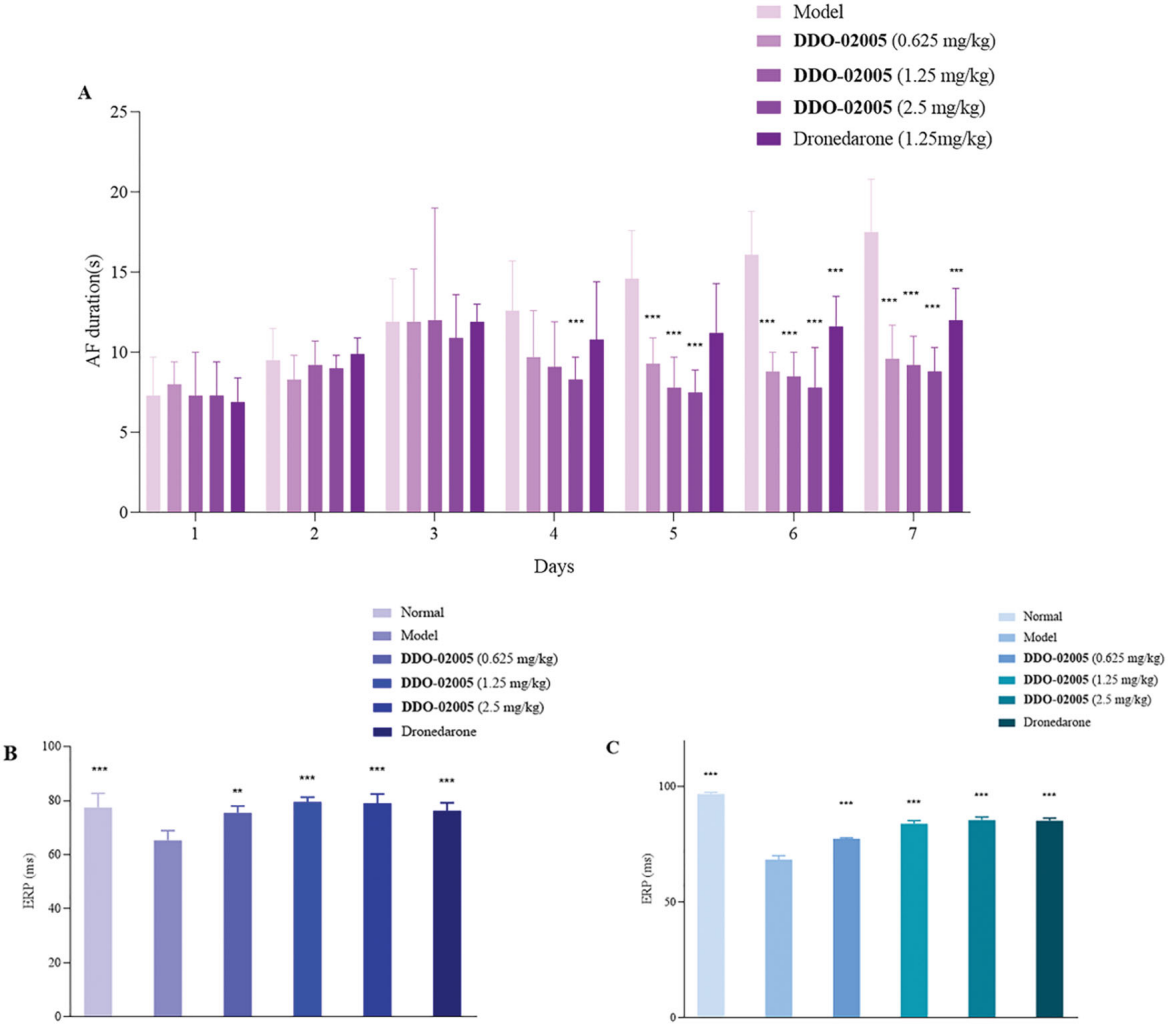
Effect of compound **DDO-02005** and dronedarone on (A) atrial fibrillation, (B) atrial ERP and (C) ventricle ERP. Values of *p < 0.05, **p < 0.01 and ***p < 0.001 were considered statistically significant.

The results showed that during Day1 – Day3 (model-creation), there was no significant difference between model group and treatment groups. The durations of AF in rats treated with **DDO-02005** (from Day 4 to Day 7) were shortened significantly. Starting from the fifth day, the effect of **DDO-02005** on the shortening of atrial fibrillation time showed a significant concentration-response relationship. As shown in Figure 4(B,C), the AERP and VERP of AF rats treated with **DDO-02005** were increased to normal level, respectively, at the dose of 2.5 mg/kg. It’s worth noting that the AF therapeutic effect of **DDO-02005** is better than that of dronedarone.

### Effect of DDO-02005 on rat arrhythmia induced by aconitines

Aconitine is a neurotoxin with strong cardiotoxicity^26^^,^^27^ which can promote the opening of L-type calcium channels on rat ventricular myocyte membranes, increase calcium influx, cause cytoplasmic calcium overload, and lead to arrhythmia^9^^,^^28^.

**Figure 5.**
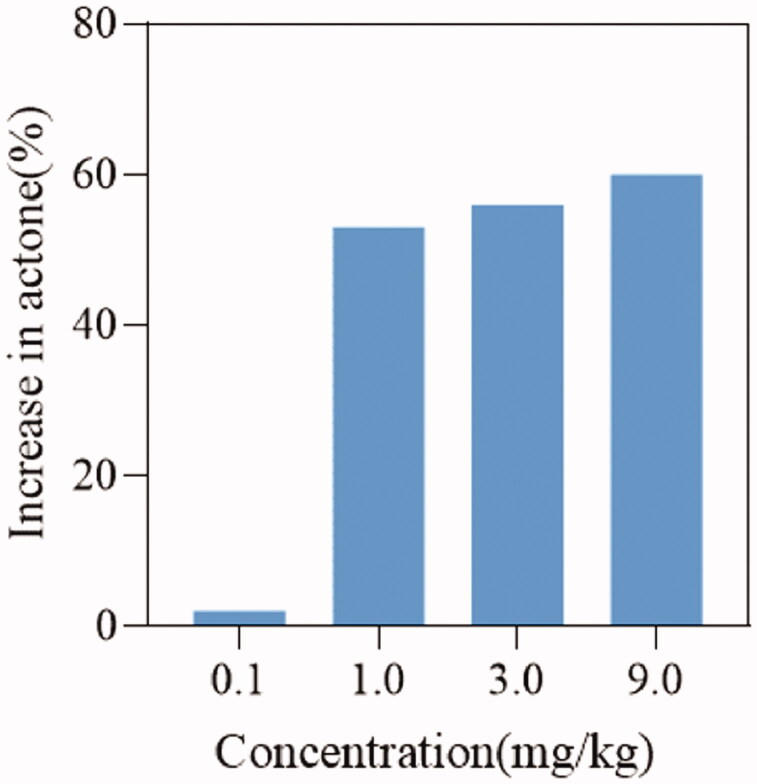
**DDO-02005** inhibits the arrhythmia induced by aconitine in rats.

To verify the effect of **DDO-02005** on arrhythmia inhibition, we established an aconitine-induced arrhythmia model in 30 rats as previously described^29^. After intubation, rats in each group were given intravenous physiological saline, and four different concentrations (0.1, 1, 3, and 9 mg/kg) of **DDO-02005** (treatment group). Taking the concentration of aconitine required to cause atrial fibrillation in the model group as a control, the concentration of aconitine increased by 2, 53, 56, and 60%, respectively. The results turned out that the compound **DDO-02005** can effectively combat the arrhythmogenic toxicity of aconitine (Figure 5).

### Pharmacokinetic (PK) study of DDO-02005

Single-dose PK studies were then performed with beagle dogs at 1 mg/kg by intravenous injection (i.v.) and 1.25 mg/kg by oral administration (p.o.), the results were summarised in Table 2. **DDO-02005** achieved the maximum plasma concentration (*C*_max_) of 1.274 μg/L, the elimination half-life (*t*_1/2_) of 6.245 h. In addition, **DDO-02005** showed a plasma clearance (CL) of 5.834 L/h/kg after the intravenous injection. Overall, the pharmacokinetic properties of **DDO-02005** need to be further optimised.

**Table 2.** Pharmacokinetic parameters regarding lead compound **DDO-02005** (mean ± SD, *n* = 6)

Parameter	*i.v.*	*p.o.*
Dose (mg/kg)	1.00	1.25
t_1/2_ (h)	3.23 ± 1.07	6.25 ± 2.40
*C*_max_ (μg/L)	90.23 ± 28.83	1.27 ± 0.40
AUC_(0-t)_ (μg/L*h)	178.42 ± 39.33	4.41 ± 0.69
CL (L/h/kg)	5.83 ± 1.44	36.51 ± 2.54

### Preliminary safety evaluation of DDO-02005

**Figure 6.**
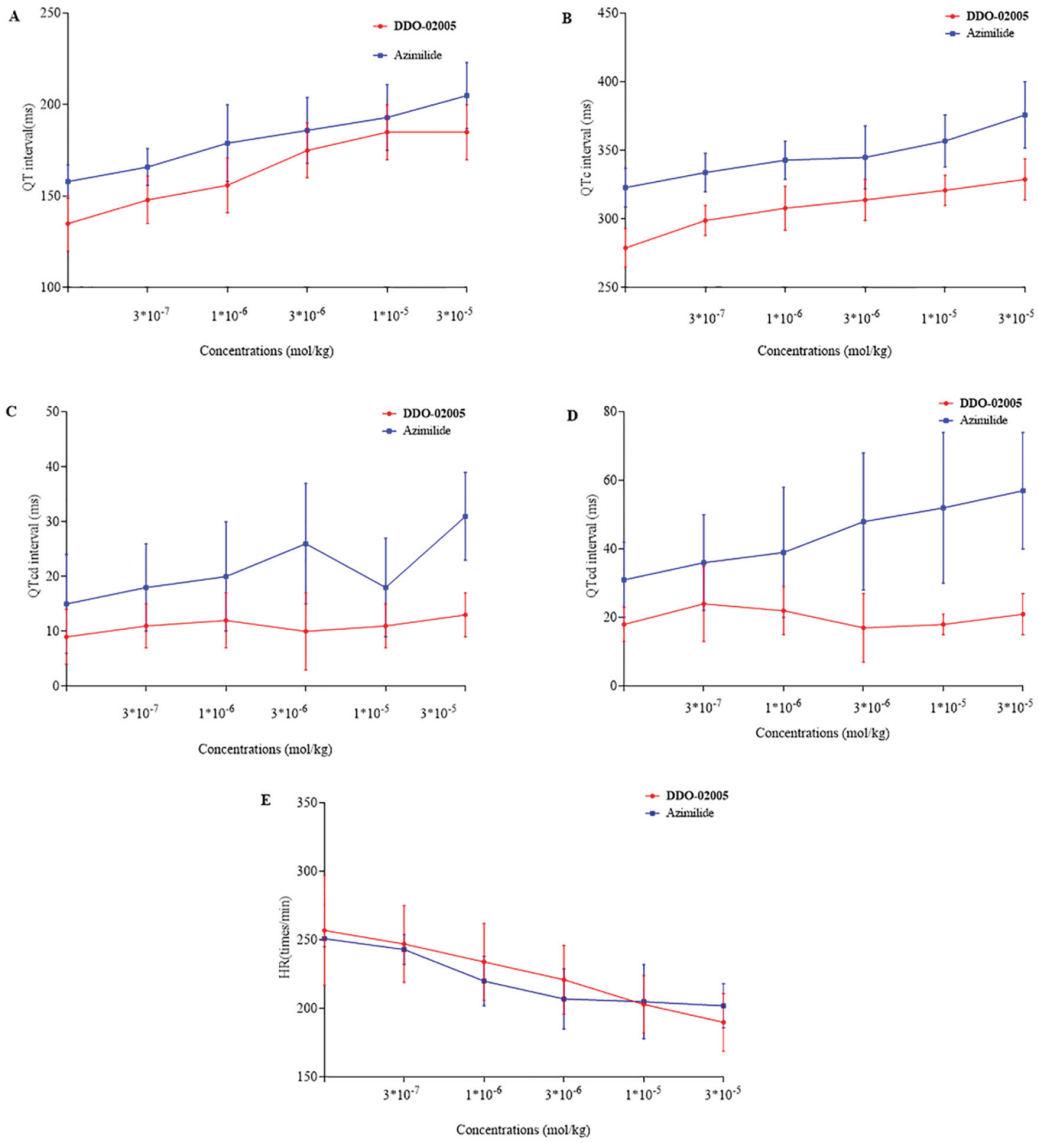
Cardiovascular parameters of guinea pigs after treated with compound **DDO-02005** (red) and Azimilide (blue), (A) QT interval. (B) QT_c_ interval. (C) QT_d_ interval. (D) QT_cd_ interval. (E) heart rate (HR). The values shown are the mean ± SD (*n* = 6).

We used a 12-lead electrocardiogram to compare preliminary safety between compound **DDO-02005** and Azimilide. As shown in Figure 6, though there were no statistically significant differences between **DDO-02005** and Azimilide in prolonging QT interval (Figure 6(A)), QT_c_ interval (Figure 6(B)), and heart rate (Figure 6(E)), **DDO-02005** had less effect on QT dispersion (Figure 6(C)) and QT_cd_ (Figure 6(D)) on guinea pigs. The heart rate of guinea pigs slowed down obviously when **DDO-02005** was used, suggesting that it is less likely to cause arrhythmia than Azimilide. All of these figures determined that **DDO-02005** is safer than Azimilide.

## Conclusion

In this study, we designed and synthesised a series of arylmethylpiperidines derivatives modified by **DDO-02001**, most of which showed effectively Kv1.5 inhibitory activities. Especially, **DDO-02005** showed excellent inhibition effect of Kv1.5 with IC_50_ of 0.72 μM. It displayed good anti-AF effect in CaCl_2_-ACh AF model and effective anti-arrhythmic activity caused by aconitine. The preliminary safety of compound **DDO-02005** was better than Azimilide showed by 12-lead electrocardiogram.

Overall, the most potent compound **DDO-02005**, which prominently inhibited Kv1.5 channel and alleviated symptom of arrhythmia with good bioavailability, in addition to being worthy of further pharmacological investigation, may be considered as a lead compound for further optimisation of Kv1.5 channel inhibitors.

## Supplementary Material

Supplemental Material
