# Supplementary material for: Design, synthesis, and biological evaluation of arylmethylpiperidines as Kv1.5 potassium channel inhibitors
Source: J Enzyme Inhib Med Chem. 2022 Jan 10;37(1):462–71. doi: 10.1080/14756366.2021.2018683 (PMC8757610; doi:10.1080/14756366.2021.2018683)
Supplement: Supplemental Material [file IENZ_A_2018683_SM7428.pdf]

<色谱图>

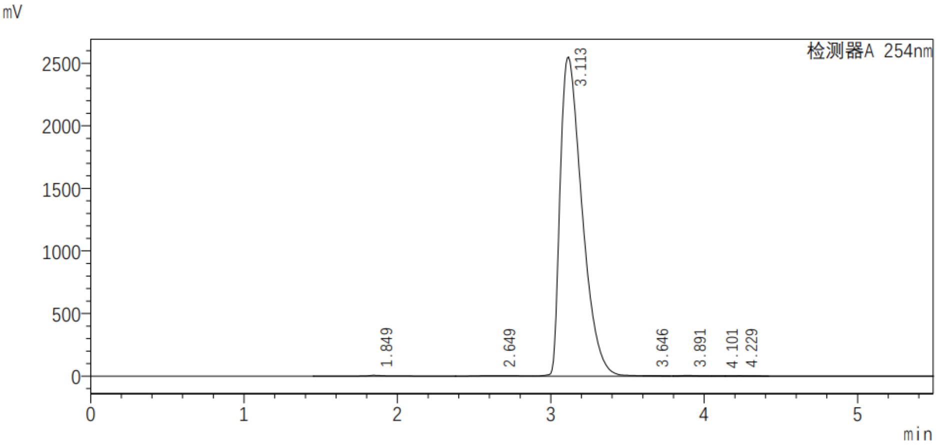

<峰表>

检测器A 254nm

峰号	保留时间	面积	高度	面积%	高度%	理论塔板数(USP)
1	1.849	47365	6211	0.186	0.242	1874
2	2.649	55485	2746	0.218	0.107	441
3	3.113	25339089	2549853	99.429	99.396	2180
4	3.646	4124	721	0.016	0.028	8749
5	3.891	23869	3706	0.094	0.144	7006
6	4.101	1043	264	0.004	0.010	--
7	4.229	13531	1857	0.053	0.072	6585
总计		25484507	2565357	100.000	100.000	

DDO-02002

<色谱图>

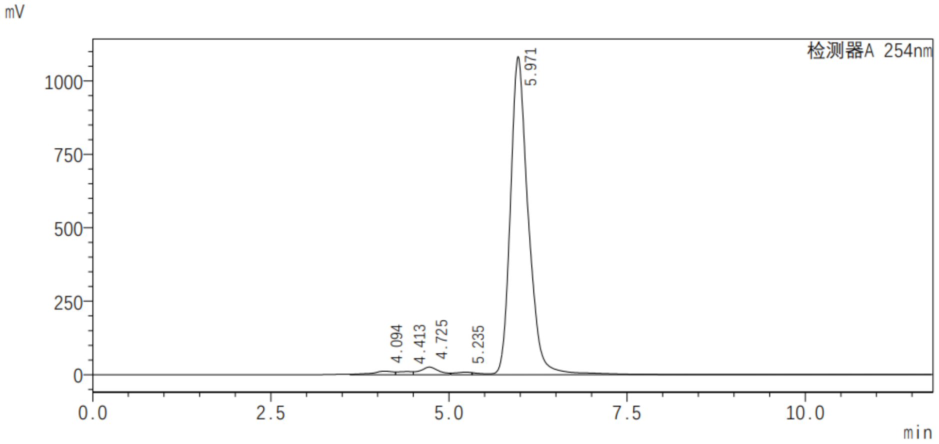

<峰表>

检测器A 254nm

峰号	保留时间	面积	高度	面积%	高度%	理论塔板数(USP)
1	4.094	244333	11728	1.220	1.030	450
2	4.413	150896	10614	0.754	0.932	80
3	4.725	456435	25759	2.280	2.262	1611
4	5.235	129741	8620	0.648	0.757	896
5	5.971	19037815	1082222	95.098	95.020	2825
总计		20019220	1138944	100.000	100.000	

DDO-02003

<色谱图>

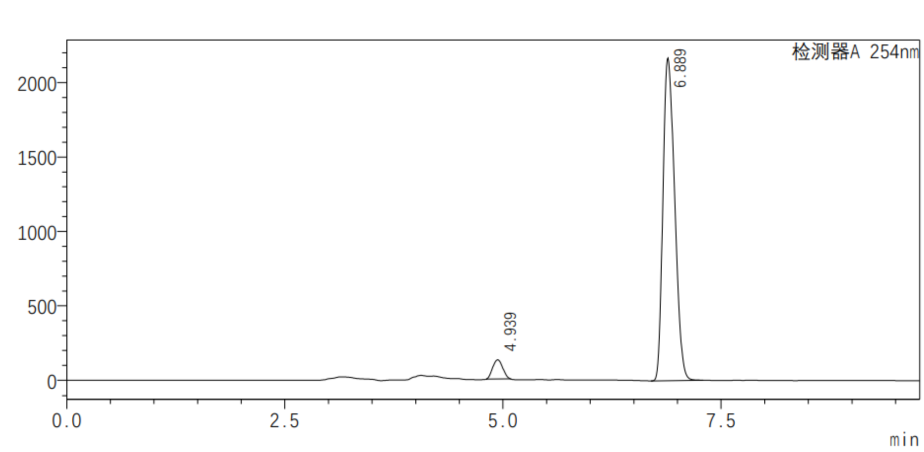

<峰表>

检测器A 254nm

峰号	保留时间	面积	高度	面积%	高度%	理论塔板数(USP)
1	4.939	1031760	129013	4.818	5.615	7845
2	6.889	20383636	2168453	95.182	94.385	11253
总计		21415396	2297466	100.000	100.000	

DDO-02004

<色谱图>

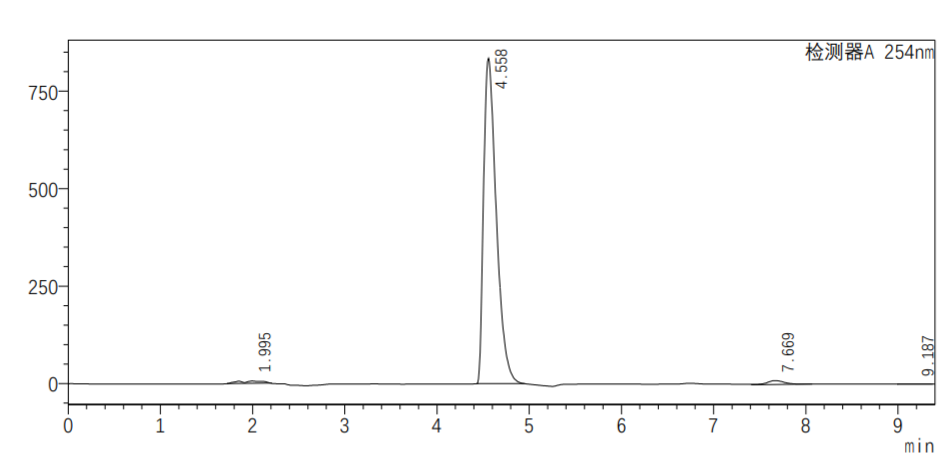

<峰表>

检测器A 254nm

峰号	保留时间	面积	高度	面积%	高度%	理论塔板数(USP)
1	1.995	112972	6096	1.373	0.717	607
2	4.558	7981293	833578	96.998	98.096	4949
3	7.669	132963	9985	1.616	1.175	8213
4	9.187	1115	97	0.014	0.011	13169
总计		8228343	849756	100.000	100.000	

DDO-02005

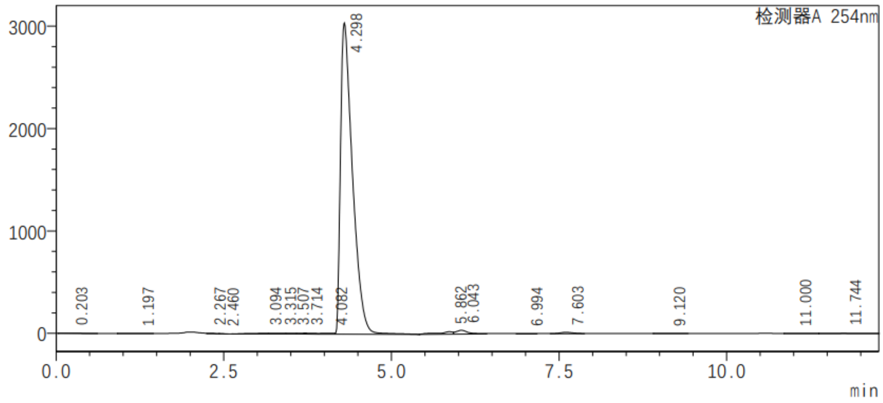

<峰表>

检测器A 254nm

峰号	保留时间	面积	高度	面积%	高度%	理论塔板数(USP)
1	0.203	12146	691	0.031	0.022	3
2	1.197	21169	845	0.053	0.027	--
3	2.267	39875	5568	0.100	0.176	66
4	2.460	1311	386	0.003	0.012	8510
5	3.094	132219	6261	0.333	0.198	3
6	3.315	105118	7154	0.265	0.226	38
7	3.507	67192	7278	0.169	0.230	22
8	3.714	156005	9355	0.393	0.295	337
9	4.082	121948	8972	0.307	0.283	127
10	4.298	37820513	3038741	95.272	95.928	2623
11	5.862	370274	24081	0.933	0.760	2220
12	6.043	514781	38055	1.297	1.201	4716
13	6.994	89290	4926	0.225	0.156	13
14	7.603	219935	14052	0.554	0.444	6175
15	9.120	2359	146	0.006	0.005	7736
16	11.000	8675	480	0.022	0.015	5048
17	11.744	14569	733	0.037	0.023	8566
总计		39697380	3167724	100.000	100.000	

DDO-02006

<色谱图>

mV

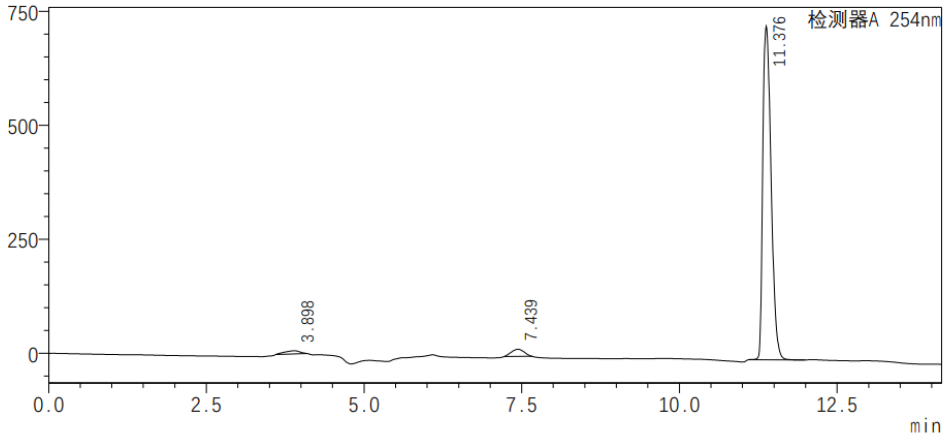

<峰表>

检测器A 254nm

峰号	保留时间	面积	高度	面积%	高度%	理论塔板数(USP)
1	3.898	124473	7151	1.763	0.948	966
2	7.439	219462	15585	3.108	2.066	5666
3	11.376	6716347	731465	95.129	96.985	34697
总计		7060281	754201	100.000	100.000	

DDO-02007

<色谱图>

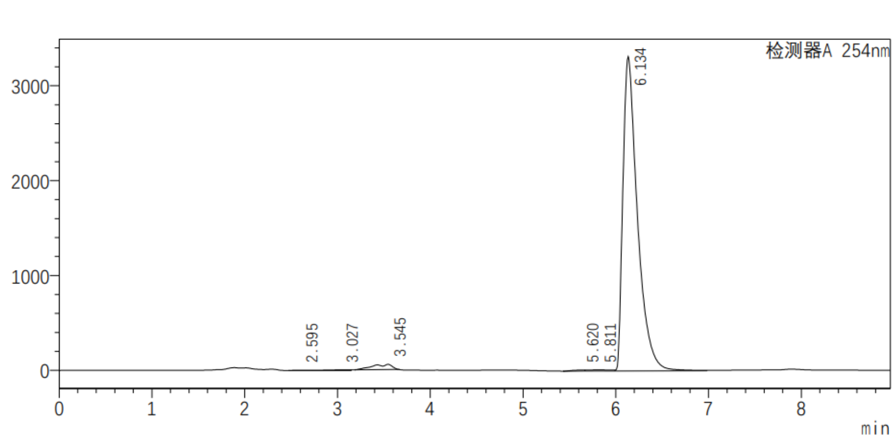

<峰表>

检测器A 254nm

峰号	保留时间	面积	高度	面积%	高度%	理论塔板数(USP)
1	2.595	27913	2530	0.074	0.074	577
2	3.027	153420	8797	0.409	0.259	350
3	3.545	811965	54859	2.166	1.612	1459
4	5.620	102272	10285	0.273	0.302	197
5	5.811	217161	12956	0.579	0.381	710
6	6.134	36168669	3312659	96.498	97.371	7309
总计		37481400	3402086	100.000	100.000	

DDO-02008

<色谱图>

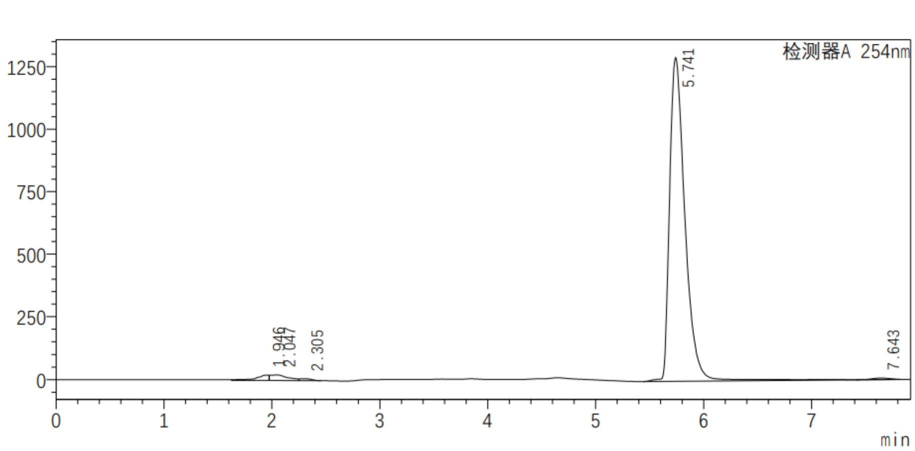

<峰表>

检测器A 254nm

峰号	保留时间	面积	高度	面积%	高度%	理论塔板数(USP)
1	1.946	187307	21331	1.388	1.581	20
2	2.047	251368	22350	1.863	1.656	44
3	2.305	56356	7524	0.418	0.558	135
4	5.741	12923273	1292246	95.796	95.751	7800
5	7.643	72067	6140	0.534	0.455	8998
总计		13490371	1349591	100.000	100.000	

DDO-02009

<色谱图>

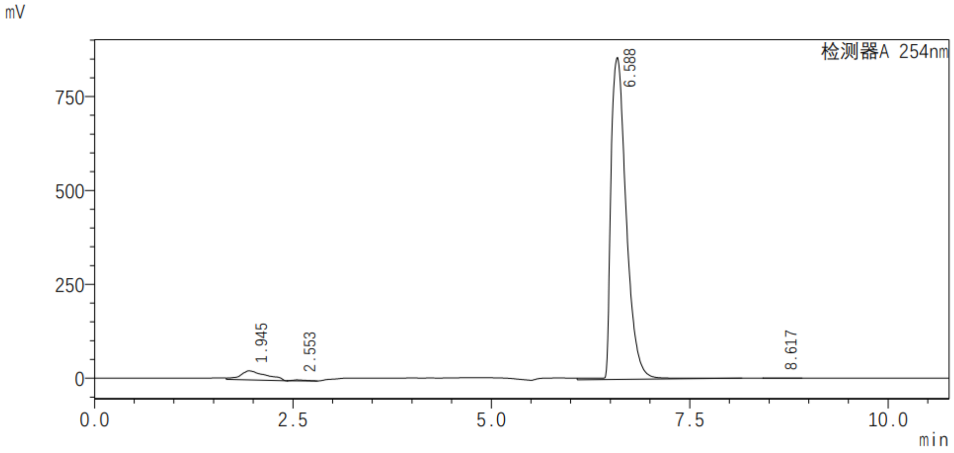

<峰表>

检测器A 254nm

峰号	保留时间	面积	高度	面积%	高度%	理论塔板数(USP)
1	1.945	547791	24057	4.530	2.724	140
2	2.553	25632	2333	0.212	0.264	999
3	6.588	11516897	856509	95.245	96.999	6174
4	8.617	1521	107	0.013	0.012	7685
总计		12091841	883006	100.000	100.000	
